# Analysis of salivary steroid hormones in boys with autism spectrum disorder

**DOI:** 10.1186/s12888-023-04586-2

**Published:** 2023-02-14

**Authors:** Qing He, Ying Wang, Zhichao Liu, Jinrong Xia, Heng Yin, Zhongqing Qiu, Hui Wang, Wenming Xu, Zhe Xu, Jiang Xie

**Affiliations:** 1 Department of Pediatrics, School of Clinical Medicine, Southwest Medical University, Luzhou, 646000 China; 2 Guangyuan Central Hospital, Guangyuan, 628000 China; 3 Deyang Jingyang Maternal and Child Health Care and Family Planning Service Center, Deyang, 618000 China; 4 Chengdu Third People’s Hospital, Qinglong Street, Qingyang District, Chengdu, 610031 Sichuan China; 5 West China Second University Hospital, Sichuan University, Chengdu, 610041 China

**Keywords:** Autism spectrum disorder, Cortisol, DHEA, Pregnenolone, Child behavior checklist, Autism behavior checklist, Social responsiveness scale, Repetitive behavior scale

## Abstract

**Background:**

Autism spectrum disorders (ASD) is a neurodevelopmental disorder with high incidence rate and difficult diagnosis. The purpose of this study was to explore whether salivary cortisol, dehydroepiandrosterone (DHEA) and pregnenolone can be used as biomarkers of ASD children.

**Methods:**

The saliva samples of 55 boys with ASD were collected as the experimental group, and the saliva samples of 24 neurotypical boys were collected as the control group. The Child Behavior Checklist (CBCL), Autism Behavior Checklist (ABC), Social Responsiveness Scale (SRS), Repetitive Behavior Scale (RBS) were used to assess the severity of symptoms in boys with ASD. Cortisol, DHEA and pregnenolone concentrations in saliva were measured using an ABSSCIEX QTRAP® 6500 + LC/MS/MS system. SPSS 23.0 was used for statistical analysis. Comparisons between the two groups which conform to normal distribution were performed by T-test, and those which don’t conform to normal distribution were performed by Mann–Whitney U test. Correlation analysis between two variables was performed using Spearman's correlation analysis. Receiver operating characteristic curve (ROC) analysis was performed to evaluate the discriminatory sensitivity of each hormone between ASD and normal control groups. Logistic regression models were used to analyze whether DHEA and salivary pregnenolone can be used as a biomarker of ASD.

**Results:**

There were no significant differences in age, and weight between the ASD group and the normal control group. The ABC, SRS, RBS and CBCL scale scores in the ASD group were significantly higher than those in the normal control group. The salivary DHEA and pregnenolone concentrations in the ASD group were significantly higher than those in the normal control group, but there was no significant difference in cortisol. Spearman's correlation analysis showed that only pregnenolone associated with ABC. Logistic regression model analysis suggested that pregnenolone in saliva was an independent predictor of ASD. ROC analysis found that pregnenolone had good discrimination sensitivity between ASD and normal controls.

**Conclusion:**

Gave salivary preoperative a space for utilization as biomarker as number of cases are limited to this high expectation.

## Background

According to the 5th edition of the diagnostic and statistical manual of mental disorders (DSM-5), autism spectrum disorders (ASD) is a neurodevelopmental disorder characterized by social interaction and communication disorders, narrow interests, stereotyped behavior and abnormal perception [1]. Neurodevelopmental changes in ASD have been reported to affect not only cognitive abilities, social brain, and other neural structures but also other major physiological systems such as immune, endocrine, and gut microbiota systems [2]. Over the past few years, the incidence of ASD has increased markedly, with approximately 1 in 68 children suffering from ASD, severely affecting the quality of life of individuals as well as caregivers and families, resulting in a heavy psychological and economic burden on families and society [2–4]. The heterogeneity of the symptomatic presentation of ASD, the lack of biomarkers and the evolving diagnostic criteria all create unique challenges in the diagnosis of ASD [5, 6]. Therefore, finding better diagnostic tools is of great significance for ASD early diagnosis and treatment.

Several studies have shown that ASD is more common in males than in females, which may be caused by androgens [7]. Human and animal studies have shown that androgen exposure results in decreased social function [7, 8]. It has been reported that the risk of ASD may stem from increased exposure to androgens (e.g., testosterone) in the prenatal period [7, 9]. At the same time, studies have also reported that postpartum androgens have persistent but impermanent effects on the human brain and cognition [10]. For example, there is a trend toward increased salivary androgen (androsterone and its polar conjugates) levels in prepubertal ASD children [11]. Pregnenolone can be converted to androstenediol via HSD3B and androstenedion is further processed to testosterone using AKRIC3 [12]. In addition, pregnenolone is a precursor of glucocorticoids [13]. Studies have shown that pregnenolone is hydroxylated at the 17th position and enters the glucocorticoid series, and the cleavage of the glucocorticoid side chain can generate androgens [14]. Corticosterone is widely regarded as the major glucocorticoid produced in amphibians [15]. Therefore, pregnenolone, DHEA, and cortisol are key hormones in the synthesis of androgens. In addition, pregnenolone, dehydroepiandrosterone (DHEA) as well as cortisol were demonstrated to exist in the serum of children with ASD [12, 16, 17]. At present, the levels of pregnenolone, DHEA, and cortisol in the saliva of children with ASD and whether they could be a diagnostic marker for children with ASD need to be further explored.

The child behavior checklist (CBCL) is one of the screening scales for assessing children's emotional and behavioral problems and is widely used to assess, collect, and rate children's internalizing and externalizing behavioral problems [18, 19]. The Autism Behavior Checklist (ABC) is a 57-item scale commonly used in clinic to characterize the behavior of children aiming to screen and diagnose ASD [20, 21]. The social responsiveness scale (SRS) is a rating scale that assesses social, communication, and repetitive behaviors associated with ASD [22]. The repetitive behavior scale (RBS) is primarily used to assess repetitive stereotypic behaviors in individuals with ASD, which is not limited to the pediatric population, and involves a wide range of behavioral items. It is more clinically applicable [23] since it is relatively easy to assess ASD. These scales are all widely used in clinical studies and are a broad-spectrum assessment tool for ASD, which can more accurately assess common behavioral problems and severity in children with ASD with good reliability and validity [23–26]. In this study, we assessed ASD severity by CBCL, ABC, SRS, and RBS.

Serum is generally considered as the "gold standard" for steroid analysis because it is thought that it can detect more biologically active compounds than saliva. However, some studies have found that some salivary steroids, such as testosterone, are highly correlated with serum steroids [27]. In addition, saliva can be collected non-invasively and non-stressfully, which is especially important for children with autism who are very vulnerable to stress. It is well known that gender has a great influence on steroid hormones. Therefore, in this study, in order to exclude the influence of gender on the results, we collected saliva samples of boys as specimens to explore the differences of salivary cortisol, DHEA and pregnenolone levels between boys with or without ASD and the relationship between salivary cortisol, DHEA and pregnenolone levels and CBCL, ABC, SRS, and RBS scale scores. Also, we investigated whether salivary cortisol, DHEA, and pregnenolone can be biomarkers for ASD.

## Methods

### Design

The present study was a descriptive study that employed appropriate scales to assess the degree of validation in boys with ASD. ABSCIEX QTRAP® 6500 + LC/MS/MS was used to test cortisol, DHEA, and pregnenolone in saliva samples. A series of statistical methods were used to analyze whether salivary cortisol, DHEA, and pregnenolone could be diagnostic markers for ASD.

### Experimental subjects

A total of 55 ASD boys as experimental group and 24 normal boys as control group. The boys with ASD were recruited from several rehabilitation institutions including the inpatient department of Jingyang maternal and child health hospital, Deyang, Sichuan, China, the outpatient clinic of Guangyuan Central Hospital, and the rehabilitation center for disabled people in Guangyuan from January 2020 to May 2022. All subjects were less than 8 years old. The control group was obtained from boys attending outpatient clinics of Guangyuan Central Hospital, Sichuan Province, and kindergartens of the organs of Guangyuan City. Inclusion criteria for control group: ① under 8 years old; ② No secondary sexual characteristics were present. Exclusion criteria: ① sexual dysplasia; ② Tumor; ③ Developmental delay, mental retardation, epilepsy and other neurological and psychiatric disorders. Inclusion criteria for boys with ASD: ①Boys diagnosed with ASD according to the DSM-5 diagnostic criteria and diagnosed by the Autism Diagnostic Observation Schedule 2nd revision (ADOS- 2). ②Boys with organic heart disease and abnormal sexual development were excluded; ③Boys with Informed consent. The study obtained the written informed consent from the boys’s parents or legal guardians. This study was carried out in accordance with the declaration of Helsinki, and reviewed and approved by the human ethics committee of Chengdu Third People's Hospital, Ethics No.: Chengdu Sanyuan Lun [2020] No. S-112.

### CBCL scale

The CBCL scale is a short, standardized questionnaire which was designed to identify social, behavioral, and emotional problems in children with ASD [28]. The CBCL scale consists of depression, somatic complaints, social withdrawal, aggression, defiant, compulsive, schizotypal, and poor interaction scores [20]. The scale was filled by children’s parents for more than half a year until the next by professionals based on the child's performance for almost half a year. The scored items were scored additively to obtain a total score for behavioral problems [18].

### ABC scale

The ABC scale consists of sensory, communicative, somatic, linguistic, physiological self-care scores. A total of 57 behavioral traits of children with autism were included. It can be used for people between 2 months and 28 years of age [20]. Completed by the parents based on the child's performance at the time of the physical examination. Scores of ≥ 67 were considered as ASD children.

### SRS scale

The SRS scale consists of social perception, social cognition, social communication, social motivation, behavioral approach scores. A total of 65 items were included. Completed by the parents based on the child's performance at the time of the physical examination. The higher the total score, the more severe the social impairment.

### RBS scale

The RBS scale consists of stereotypic behavior, self-injurious behavior, compulsive behavior, ritualistic behavior, fixed behavior, and restricted behavior scores. A total of 57 behavioral traits were included. It can be used as an autism screening scale for children between 2 and 14 years of age [29]. Completed by the parents based on the child's performance at the time of the physical examination. Higher scores represent more pronounced autistic features.

### Physical examination and evaluation criteria

The height and weight of all enrolled boys were collected, and the weight-for-age Z-score (WAZ) and the height-for-age Z-score (HAZ) were calculated according to the 2006 World Health Organization's reference standards for height and weight for children aged 0–18 years. Z score = (measured value—median of reference standard) / standard deviation of reference standard.

### Saliva sample collection and determination of hormone concentration

Saliva collection: all subjects refrained from consuming alcohol, coffee, beverages within 12 h before performing saliva collection. The following morning, brushing teeth at bedtime at night before collection, fasting in the morning, and about 2 ml of saliva was collected with a disposable saliva collector at resting state and put into -80℃ for freezing. Salivary hormone testing: Cortisol, DHEA and pregnenolone in saliva samples were detected by ABSSCIEX QTRAP® 6500 + LC/MS/MS system, data was collected by Analyst 1.6.2 software, and quantitative analysis was performed by Multiquant software.

### Statistical analysis

Sample size was calculated using G-Power software. We aimed at 21 cases per group, as this would yield sufficient power (80%) at the α = 0.05 level to detect a large effect size (d = 0.8) in the Wilcoxon-Mann–Whitney test (two groups) [30]. The sample size we actually collected was 24 cases in the Ctrl group and 55 cases in the ASD group. Therefore, our sample size was reasonable.

All data in this paper were statistically analyzed using SPSS 23.0. The normality of the data distribution was assessed using the Kolmogorov–Smirnov test. Normally distributed data are presented as mean ± standard deviation, while non-normally distributed data are presented as median and their respective 25% and 75% boundaries. The comparison between the two groups conformed to the normal distribution using the T test. The Mann–Whitney U test was used if the comparison which didn’t conform to the normal distribution. The correlation analysis was performed by Spearman correlation coefficient(r). ROC curve analysis discriminates sensitivity. Logistic regression models analyzed whether salivary pregnenolone could be a biomarker for ASD. Statistical difference was indicated by *p* < 0.05. The figure in this article is drawn using Graphpad Prism8.0.2.

## Results

### Demographic characteristics

Demographic comparison results showed that there was no significant difference in age, height and weight between the ASD group and the normal control group (all *P* > 0.05). The results are shown in Table 1.

**Table 1:** Comparison of general sociodemographic characteristics of the study subjects

	Ctrl (*n* = 24)	ASD (*n* = 55)	*P*
Age	5.17 (4.58, 5.54)	5.08 (4.50, 5.75)	0.987
HAZ	0.15 (-0.07, 0.57)	0.24 (-0.44, 0.53)	0.782
WAZ	0.12 ± 0.84	0.33 ± 0.87	0.326

### The scores of each scale between the normal control group and the ASD group

All boys with ASD and normal control groups were evaluated by ABC, SRS, RBS and CBCL scales, and the score results are shown in Table 2. It was found that the CBCL, ABC, SRS and RBS scale scores of the ASD group were significantly higher than those of the normal control group ( All *P* < 0.05).

**Table 2:** Comparison of scores on each scale between the normal control and ASD groups

	Ctrl	ASD	*P*
ABC	0.00 (0.00, 5.50)	54.00 (37.00, 74.00)	< 0.001
SRS	26.50 (24.00, 40.50)	89.00 (62.00, 103.00)	< 0.001
RBS	3 (1.00, 9.75)	17.00 (10.00, 31.00)	< 0.001
CBCL	10.00 (6.00, 40.75)	45.00 (31.00, 66.00)	< 0.001

*ABC* Autism Behavior Checklist, *SRS* Social Responsiveness Scale, *RBS* Repetitive Behavior Scale, *CBCL* Child Behavior Checklist

### Comparison of hormone concentrations in saliva between normal control group and ASD group

Differences in the concentrations of cortisol, DHEA and pregnenolone in the saliva of ASD boys and normal control boys were analyzed by T-test or Mann–Whitney U test. In saliva, cortisol concentrations were not significantly different between ASD and normal controls, whereas DHEA and pregnenolone were significantly higher in ASD (Table 3, all *P* < 0.05).

**Table 3:** Salivary cortisol, DHEA, and pregnenolone levels in boys with ASD and normal controls

	Ctrl	ASD	*P*
Cortisol	798.70 (622.26, 1176.80)	864.04 (552.71, 1152.89)	> 0.05
DHEA	20.33 (6.96, 77.70)	71.99 (19.92, 166.03)	0.004
Pregnenolone	12.28 (6.62, 24.88)	29.95 (12.03, 168.06)	0.001

### Relationship between concentrations of each hormone and scores on each scale

The relationships between DHEA and pregnenolone concentrations and scores on each scale were tested by Spearman. The results showed that only pregnenolone associated with ABC (Table 4). We suggest that this phenomenon may be related to the complex etiology, genes, environment, and diet of ASD.

**Table 4:** Relationship between concentrations of each hormone and scores on each scale

	ABC		SRS		RBS		CBCL	
	r	*P*	r	*P*	r	*P*	r	*P*
DHEA	0.097	0.481	0.072	0.600	-1.51	0.270	-0.126	0.361
Pregnenolone	0.687	< 0.001***	0.016	0.909	-0.132	0.337	0.105	0.447

r correlation coefcient

^***^Correlation is signifcant at *p* < 0.001

### Boys with high pregnenolone have a higher risk of ASD

Since only pregnenolone was associated with ABC in each of the ASD rating scales, we used logistic regression models to test whether salivary pregnenolone could be a biomarker of ASD. The results showed that the association of the variable pregnenolone with ASD was statistically significant. Boys with higher pregnenolone had a higher risk of ASD (Table 5).

**Table 5:** Logistic regression model was used to analyze whether pregnenolone in saliva was an independent predictor of ASD

Variable	B	S.E	Wald	df	Sig	Exp(B)	Exp(B)	
							95%(L)	95%(U)
Pregnenolone	0.022	0.011	3.898	1	0.048	1.028	1.023	0.968
Constant	-0.33	0.362	0.008	1	0.928	0.738		

### Pregnenolone in saliva has good discrimination sensitivity between ASD and normal controls

**Fig. 1:**
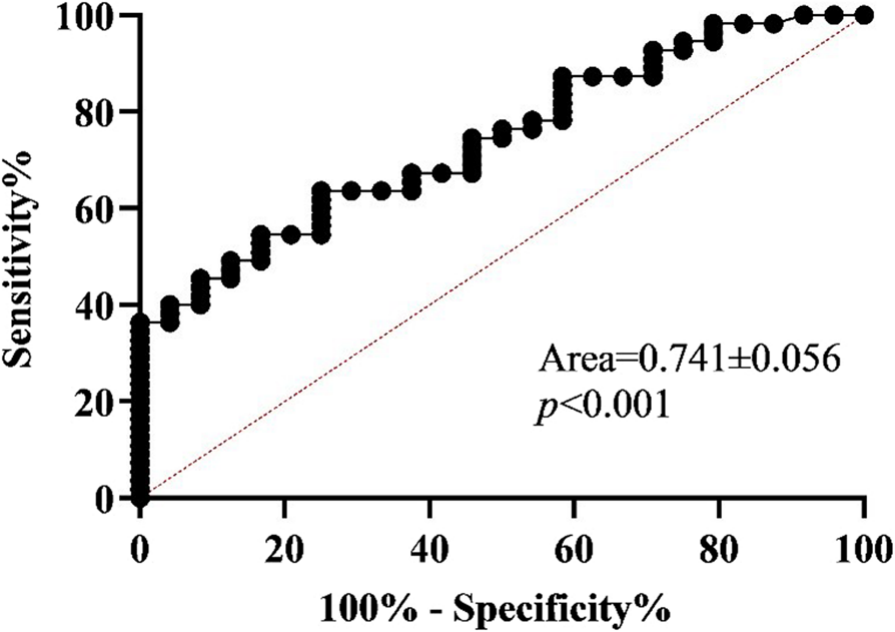
ROC curve discriminating sensitivity of ASD and Ctrl classification

Further, we performed a ROC analysis of salivary pregnenolone, and the results showed that salivary pregnenolone had good discrimination sensitivity between ASD and normal controls (Fig. 1).

## Discussion

ASD primarily occurs in early childhood, so it is called childhood autism. ages 2–5 years are the most obvious stage of ASD behavior, and the incidence of ASD in males is higher than in females [11, 31, 32]. In our study, we only investigate cortisol, DHEA and pregnenolone in saliva samples from boys to rule out gender effect on the results. Emotional dysregulation, irritability, anxiety, irritability, and aggressive behaviors or avoidant withdrawal are commonly observed during the development of ASD, severely impacting development, communication, socialization, and behavior, and life [33–35]. It is reported that only 10–35% of ASD cases have known major risk factors or established etiologies [36]. Because of the difficulty of early diagnosis of ASD, early treatment cannot be performed, leading to a poor prognosis of ASD. As early diagnosis improves the efficacy of behavioral therapies, molecular biomarkers represent an attractive approach to identify 'at-risk' populations and may contribute to the development of personalized therapies [37]. This prospect is becoming increasingly important given the rising rate of ASD diagnosis.

At present, there are no objective and reliable biomarkers for the diagnosis of ASD, which mainly rely on clinical diagnosis and standardized diagnostic scale evaluation [38, 39]. For example, Kim [40] et al. examined state and anxiety levels in adolescents with ASD by analyzing Pearson correlations between RRB, problem behavior variables, and the State/Trait Anxiety Inventory (STAI) and CBCL. Li [41] et al. used the ABC scale and the Childhood Autism Rating Scale to assess the severity of ASD. We used the CBCL, ABC, SRS and RBS scales to evaluate the behavioral problems of boys with ASD, and found that the CBCL, ABC, SRS and RBS scores in the ASD group were significantly higher than those in the normal control group.

Currently, in addition to genetics, non-genetic factors such as environment, metabolism, infection, gut microbiota, and endocrine have all been reported to be associated with the occurrence of ASD [29, 36, 42, 43]. Sex steroids, such as testosterone, are thought to be one of the biological factors associated with neurodevelopmental disorders including ASD [44]. During brain development, the expression of genes related to steroid biosynthesis increases [45]. It has been confirmed that high prenatal sex steroid levels are associated with autistic features in infants and children, meanwhile, increased circulating testosterone levels have also been shown in ASD patients during childhood or adulthood [46]. Majewska et al. [11] found increased levels of internal steroid hormones such as pregnenolone and DHEA in saliva of prepubertal children with ASD, but no significant differences in cortisol. It has also been shown that at night, cortisol levels are significantly higher in children with ASD [47, 48]. Our study found that salivary levels of dehydroepiandrosterone and pregnenolone were significantly increased in ASD patients, but cortisol was not significantly different, and only pregnenolone was significantly associated with the RBS score. The different results from previous study may be related to salivary collection time, complex etiological, genetic, environmental, and dietary differences in ASD.

## Conclusions

Salivary levels of DHEA and pregnenolone were significantly increased in ASD cases, but cortisol was not significantly different. In addition, DHEA was not significantly associated with any of the scale scores. Pregnenolone was only significantly associated with RBS scores. The follow-up study found that boys with high pregnenolone had a higher risk of ASD than those with low pregnenolone, and it had good discriminatory sensitivity for ASD. Therefore, give salivary preoperative a space for utilization as biomarker as number of cases are limited to this high expectation.

## Limitations

The small sample size, not enough variety of hormone assays, gender limitations and lack of diversity of samples are the main limitations of this paper. In addition, eating patterns, saliva collection times, and exercise among control and ASD children were not assessed and controlled, and it is unclear whether diet and sampling times have an effect on hormone levels. And the specific mechanism is still unclear. In later studies, we will continue to collect more saliva and blood samples, detect more hormone (e.g., testosterone, progesterone, etc.) levels, and further explore the specific mechanism.

## Data Availability

The datasets used and/or analyzed during the current study are available from the corresponding author on reasonable request.

## Abbreviations

ASD: Autism spectrum disorders
DHEA: Dehydroepiandrosterone
CBCL: Child Behavior Checklist
ABC: Autism Behavior Checklist
SRS: Social Responsiveness Scale
RBS: Repetitive Behavior Scale
ROC: Receiver operating characteristic curve
